# Universal versus risk– factor–based screening for gestational diabetes mellitus: findings from a large–scale cohort study

**DOI:** 10.3389/fendo.2026.1918348

**Published:** 2026-08-13

**Authors:** Mohammadmahdi Fallah, Maryam Mousavi, Arefeh Tabashiri, Faegheh Firouzi, Fereidoun Azizi, Fahimeh Ramezani Tehrani, Samira Behboudi-Gandevani

**Affiliations:** 1 Reproductive Endocrinology Research Center, Research Institute for Endocrine Molecular Biology, Research Institute for Endocrine Sciences, Shahid Beheshti University of Medical Sciences, Tehran, Iran; 2 Endocrine Research Center, Research Institute for Endocrine Disorders, Research Institute for Endocrine Sciences, Shahid Beheshti University of Medical Sciences, Tehran, Iran; 3 Foundation for Research & Education Excellence, Vestavia Hills, AL, United States; 4 Faculty of Nursing and Health Sciences, Nord University, Bodø, Norway

**Keywords:** adverse pregnancy outcome, gestational diabetes mellitus, LASSO regression analyses, risk factors, targeted screening, universal screening

## Abstract

**Objectives:**

Gestational diabetes mellitus (GDM) carries significant feto-maternal risks. While early diagnosis and treatment effectively mitigate these adverse outcomes, the most effective strategy for screening and detection remains a subject of ongoing debate.

**Methods:**

A secondary analysis of data from a large trial involving 35,430 women was conducted to estimate the proportion of GDM cases missed by targeted screening, compare maternal and neonatal outcomes between identified and potentially overlooked pregnancies, and identify maternal risk factors most strongly associated with adverse pregnancy outcomes using multivariable logistic and least absolute shrinkage and selection operator (LASSO) regression analyses.

**Results:**

Approximately 11% of women not recommended for testing under a risk factor–based targeted screening strategy would have remained undiagnosed. The full 11-factor model and the simplified three-factor LASSO model, comprising increasing maternal age, obesity, and prior GDM, showed comparable predictive performance. Women with both GDM and maternal risk factors had the highest likelihood of adverse outcomes, whereas women with GDM but without maternal risk factors had lower complication rates than non-GDM women with maternal risk factors.

**Conclusion:**

A simplified three-factor screening model demonstrated acceptable predictive performance and may provide a practical alternative in resource-limited settings. Furthermore, the coexistence of GDM and maternal risk factors was associated with increased clinical vulnerability, highlighting the need for closer surveillance and targeted management of these pregnancies.

## 1 Introduction

Gestational diabetes mellitus (GDM) affects 4% to 12% of pregnancies and carries significant risks for both mother and newborn (1–4). While prompt diagnosis and treatment effectively mitigate these adverse outcomes, the optimal screening strategy remains highly debated (5, 6). Universal screening maximizes detection and early intervention, but it places a heavy financial and operational burden on healthcare systems and risks over-medicalizing pregnancies by labeling more women with a GDM diagnosis. Conversely risk-factor-based or targeted screening is more cost-effective and frequently utilized in resource-limited settings (7–10). However, relying only on established maternal risk factors, including advanced age, obesity, polycystic ovary syndrome, and a family history of diabetes, might miss a significant number of affected pregnancies (11). Pregnancy is accompanied by substantial physiological and metabolic adaptations, which may contribute to the development of GDM even in the absence of conventional risk factors; accordingly, an estimated 10% to 50% of GDM cases occur in women without identifiable traditional predispositions (5, 8, 12, 13). Nevertheless, women who develop GDM in the presence of established maternal risk factors may represent a particularly high-risk subgroup, with an increased likelihood of adverse maternal and neonatal outcomes (14).

A significant and unresolved issue in the literature concerns the maternal and neonatal outcomes among women whose GDM remains undetected due to targeted screening strategies. It remains uncertain whether these undiagnosed women experience adverse pregnancy outcomes comparable to those diagnosed through risk factor-based screening, or whether the absence of baseline risk factors confers a degree of protection against such complications. Addressing this uncertainty has been challenging due to considerable heterogeneity across previous studies, including differences in study design, screening strategies, diagnostic criteria, and reported outcome measures (15, 16). There are also discrepancies in the number and definitions of maternal risk factors, including age limits, BMI cutoffs, and whether obstetric history is considered (12, 13). These variations have made it difficult to directly compare outcomes and measure the true clinical cost of targeted screening.

To address this gap, we conducted a secondary analysis of data from a large population-based study involving over 35,000 pregnancies in Iran. While universal screening was used in the original study, detailed information on various maternal risk factors allowed us to determine how many GDM cases would have been missed with targeted screening strategies. The objectives of this study are to estimate the proportion of GDM cases that would be missed under a targeted screening strategy; to compare maternal and neonatal outcomes between pregnancies identified through targeted screening and those potentially overlooked; and to determine the maternal risk factors most strongly associated with adverse pregnancy outcomes. Ultimately, these findings aim to refine and optimize risk-based screening protocols for resource-limited areas.

## 2 Methods

This study approved by ethics committee of the Research Institute for Endocrine Sciences, Shahid Beheshti University of Medical Sciences, Tehran, Iran (IR.SBMU.ENDOCRINE.REC.1404.129).

### 2.1 Study design and data source

This study is a secondary analysis of data from a large cluster-randomized non-inferiority trial that took place in Iran. The main trial, registered as IRCT138707081281N1 (10, 17), aimed to compare five different screening protocols for GDM. In that trial, over 35,000 pregnant women from five regions in Iran were screened for GDM using one of five specified protocols between September 2016 and January 2019.

### 2.2 Study population

**Figure 1:**
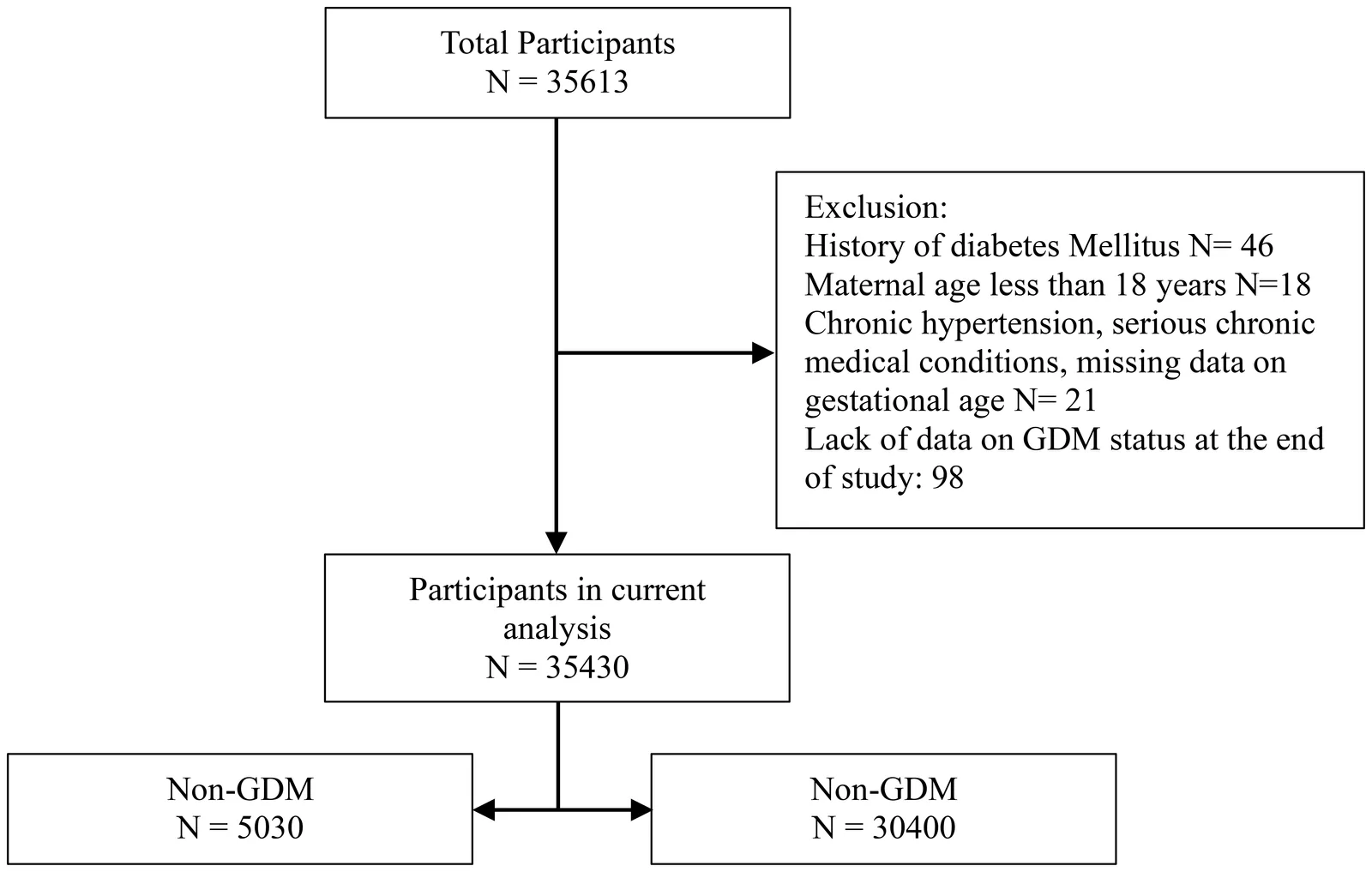
Study flowchart.

The original trial included pregnant women in their first trimester (gestational age under 14 weeks) who sought prenatal care through the public health system. Key exclusion criteria included maternal age under 18 years, pre-existing diabetes, uncertain gestational age, chronic hypertension, serious chronic medical conditions (such as asthma requiring oral glucocorticoids), and a history of bariatric surgery. For this secondary analysis, we considered all participants from the original cohort. Women with missing data on baseline risk factors, GDM diagnostic results, or pregnancy outcomes were excluded. The final analytic sample consisted of 35,430 women, as complete information was available for all eligible participants. **Figure 1** shows study flowchart.

### 2.3 GDM diagnosis

Gestational diabetes mellitus was defined according to five different diagnostic protocols (Protocol A to E). In the first trimester, GDM was diagnosed based on fasting plasma glucose (FPG) values using either a lower threshold (5.1 mmol/L [92 mg/dL]) in Protocols A and D or a higher threshold (5.6 mmol/L [100 mg/dL]) in Protocols B, C, and E, with an upper limit of 7.0 mmol/L (126 mg/dL) in all protocols. In the second trimester, Protocols A, B, and C used a one-step 75-g oral glucose tolerance test (OGTT), while Protocols D and E employed a two-step approach (50-g glucose challenge test followed by a 100-g OGTT if indicated). Diagnostic criteria for the 75-g OGTT required at least one abnormal value (Protocol A and C) or at least two abnormal values (Protocol B). For the 100-g OGTT, GDM was diagnosed when at least two plasma glucose values met or exceeded the respective thresholds. The specific protocol used for each participant was recorded and adjusted for as a covariate in the statistical analyses.

### 2.4 Risk factors assessed

We evaluated a set of 11 possible maternal risk factors for GDM based on data collected during the first prenatal visit through structured questionnaires and medical records. These factors included:

Maternal age ≥35 years; BMI ≥30 kg/m^2^; history of GDM in a previous pregnancy, chronic hypertension, prior spontaneous abortion or recurrent pregnancy loss, dyslipidemia, a history of polycystic ovary syndrome (PCOS), a history of macrosomia (birth weight >4000 g), a history of fetal anomaly, and a history of intrauterine fetal death (IUFD); and family history of diabetes in a first-degree relative.

These factors were considered both individually and collectively in modeling hypothetical targeted screening strategies.

### 2.5 Risk-factor–based screening strategies

Two simulated screening strategies were evaluated: (i) Full Risk-Factor Model (11-Factor Model), women with ≥1 of the eleven predefined risk factors were considered eligible for biochemical screening. (ii) least Absolute Shrinkage and Selection Operator (LASSO) Derived Parsimonious Model (18), was applied to all candidate maternal risk factors to identify a parsimonious subset of predictors for GDM. LASSO applies an L1 penalty to the regression coefficients, shrinking less informative predictors toward zero while retaining the most informative variables. The tuning parameter (λ), which controls the degree of shrinkage, was selected using 10-fold cross-validation. Two values were considered: λ_min, which minimizes the cross-validation error, and λ_1SE, the largest value within one standard error of the minimum error. The λ_1SE model was selected because it provides a simpler model with comparable predictive performance while reducing the risk of overfitting.

Model performance for each strategy was assessed using proportion of women screened vs. not screened, detection rate of GDM among screened women, rate of missed GDM cases among unscreened women, and area under the ROC curve (AUC).

### 2.6 Classification of outcome groups

For maternal and neonatal outcome analysis, participants were categorized into four mutually exclusive groups based on their GDM status and presence of maternal risk factors:

Group 1: No GDM and no maternal risk factors; Group 2: No GDM but ≥1 maternal risk factor; Group 3: GDM without maternal risk factors; Group 4: GDM with ≥1 maternal risk factor. Group 1 served as the reference category in all comparative analyses.

This predefined structure allowed evaluation of the independent and combined effects of GDM and maternal risk-factor burden on perinatal outcomes.

### 2.7 Maternal and neonatal outcomes

The adverse examined outcomes included: macrosomia, preterm birth, low birth weight, primary cesarean delivery, neonatal hypoglycemia, pre-eclampsia, neonatal intensive care unit (NICU) admission, and IUFD.

### 2.8 Statistical analysis

The initial analysis focused on summarizing baseline characteristics. Categorical variables were presented as frequencies and percentages [N (%)] and compared between the GDM and non-GDM groups using the Chi-Square test of independence.

Logistic regression models were used to estimate crude and multivariable-adjusted associations, adjusting for maternal age, body mass index, gestational age at enrollment, gestational age at delivery, GDM screening protocol, and mode of delivery, to examine the associations of GDM status and maternal risk factors with adverse pregnancy outcomes. These models were also used to examine the associations of GDM status and maternal risk factors (per each risk factor and multivariable model including all of these maternal risk factors). Odds ratios (ORs), along with 95% confidence intervals (CI) and p-values, were reported to determine statistical significance.

To simplify the model while retaining predictive power, LASSO regression was applied to identify the most relevant maternal risk factors associated with GDM. LASSO performs simultaneous variable selection and coefficient shrinkage by applying an L1 penalty, reducing less informative predictors toward zero and improving model generalizability.

Model tuning was performed using 10-fold cross-validation. The mean cross-validated prediction error was calculated across a sequence of penalty parameters (λ), and two optimal λ values were identified: λ_min, corresponding to the minimum cross-validated error, and λ_1_SE, representing the most parsimonious model within one standard error of the minimum error. The number of non-zero coefficients retained at each λ value was recorded to assess model complexity.

The performance of the resulting full factor model and the reduced factor model (based on LASSO) was quantified using the area under the receiver operating characteristic curve (AUC of ROC curve), calculated based on the individual risk scores derived from each model.

Statistical analyses were performed using R software (version 4.5.0). LASSO regression was implemented using the glmnet package, with the optimal penalty parameter selected by the cv.glmnet function using 10-fold cross-validation. All statistical analyses were conducted with a significance level set at α = 0.05.

## 3 Results

### 3.1 Baseline cohort characteristics

The baseline characteristics associated with the risk of GDM among 35,430 participants are summarized in **Table 1**, of whom 5,030 (14.2%) developed GDM. Women with GDM were significantly older and more likely to have a BMI ≥ 30 kg/m^2^ compared with their non-GDM counterparts (31.9% vs. 20.9% and 24.0% vs. 14.8%, respectively; *P* < 0.001 for both).

**Table 1:** Baseline GDM risk related characteristics of the cohort.

Variables	Total participants (N=35430)	GDM status N (%)		P-value
		Yes 5030 (14.2)	No 30400 (85.8)	
Age over 35 years	7711(21.8%)	1604(31.9%)	6107(20.9%)	<0.001
BMI over 30 kg/m2	5706(16.1%)	1205(24.0%)	4501(14.8%)	<0.001
History of Hypertension	177(0.5%)	50(1.0%)	127(0.4%)	<0.001
History of GDM	533(1.5%)	200(4%)	333(1.1%)	<0.001
Family history of diabetes	3489(9.8%)	654(13%)	2835(9.3%)	<0.001
History of abortion	1476(4.2%)	237(4.7%)	1239(4.1%)	0.036
History of dyslipidemia	71(0.2%)	21(0.4%)	50(0.2%)	<0.001
History of PCOS	1029(2.9%)	140(2.8%)	889(2.9%)	0.581
History of macrosomia	405(1.1%)	83(1.7%)	322(1.1%)	<0.001
History of fetal anomalia	210(0.6%)	23(0.5%)	187(0.6%)	0.177
History of IUFD	308(0.9%)	55(1.1%)	253(0.8%)	0.065

GDM, Gestational Diabetes Mellitus; BMI, Body Mass Index; PCOS, Polycystic Ovary Syndrome; IUFD, Intrauterine Fetal Death.

A higher prevalence of comorbidities and metabolic risk factors was evident among GDM cases, including history of hypertension (1.0% vs. 0.4%), previous GDM (4.0% vs. 1.1%), and family history of diabetes (13.0% vs. 9.3%), all showing statistically significant associations (*P* < 0.001). Moreover, dyslipidemia, though rare, was more frequent in women with GDM (*P* < 0.001).

Obstetric history revealed marginal but significant differences for prior abortion (4.7% vs. 4.2%, *P* = 0.036) and macrosomia (1.7% vs. 1.1%, *P* < 0.001), indicating a potential link between these reproductive outcomes and later GDM risk. Conversely, there were no significant differences in the prevalence of PCOS, fetal anomalies, or IUFD.

### 3.2 Risk factor associations with GDM via logistic regression

The associations between maternal risk factors and GDM based on logistic regression models are presented in **Table 2**. In the univariable model, maternal age over 35 years and BMI ≥ 30 kg/m^2^ were both significantly associated with an increased risk of GDM (OR = 1.86 and 1.81, respectively; (*P* < 0.001 for both). In multivariable models with all risk factors, these associations remained robust though slightly attenuated (OR = 1.69 and 1.65, *P* < 0.001). A previous history of GDM showed the strongest independent association (adjusted OR = 3.03; 95% CI 2.52–3.64; (*P* < 0.001). History of hypertension also remained significant in multivariable model (OR = 1.46; (*P* = 0.027), suggesting an independent metabolic risk relationship. Both family history of diabetes and dyslipidemia retained their significance in the multivariable model, though with reduced effect sizes (OR = 1.30 and 1.74, respectively). While history of abortion was associated with GDM in univariable analyses (OR = 1.16; *P* = 0.037), it lost significance after adjustment.

**Table 2:** Associations of maternal risk factors with GDM by Logistic regression analysis.

Variable	Model 1		Model 2	
	OR (95% CI)	P-value	OR (95% CI)	P-value
Age over 35 years	1.862 (1.744 to 1.989)	<0.001	1.695 (1.584 to 1.814)	<0.001
BMI over 30 kg/m2	1.813 (1.687 to 1.948)	<0.001	1.656 (1.538 to 1.783)	<0.001
History of GDM	3.739 (3.129 to 4.467)	<0.001	3.034 (2.528 to 3.640)	<0.001
History of Hypertension	2.393 (1.723 to 3.324)	<0.001	1.467 (1.045 to 2.058)	0.027
Family history of diabetes	1.453 (1.327 to 1.591)	<0.001	1.306 (1.189 to 1.434)	<0.001
History of abortion	1.164 (1.009 to 1.342)	0.037	0.947 (0.818 to 1.096)	0.467
History of dyslipidemia	2.545 (1.527 to 4.240)	<0.001	1.740 (1.021 to 2.966)	0.042
History of PCOS	0.950 (0.793 to 1.139)	0.581	0.840 (0.698 to 1.011)	0.065
History of macrosomia	1.567 (1.229 to 1.199)	<0.001	1.126 (0.877 to 1.447)	0.352
History of fetal anomalia	0.742 (0.481 to 1.146)	0.178	0.585 (0.374 to 0.915)	0.019
History of IUFD	1.317 ( 0.983 to 1.766)	0.065	1.142 (0.842 to 1.548)	0.394

Model 1: univariable per each specific risk factor.

Model 2: Multivariable model including all maternal risk factors.

GDM, Gestational Diabetes Mellitus; BMI, Body Mass Index; PCOS, Polycystic Ovary Syndrome; IUFD, Intrauterine Fetal Death.

### 3.3 LASSO regression risk factor selection

The results of LASSO regression applied to 11 potential maternal risk factors for GDM are presented in **Table 3**. Using the penalization parameter at λ_1_SE (the most parsimonious model within one standard error of the minimum deviance), the model identified three variables with non-zero coefficients: maternal age over 35 years, BMI ≥ 30 kg/m^2^, and a history of GDM. The predictors retained positive coefficients, indicating that increasing values were associated with higher likelihood of GDM. Specifically, the odds ratios corresponding to these coefficients were 1.16 for older age, 1.06 for obesity, and 1.11 for prior GDM, underlining their relative but modest independent contributions in the penalized model. All other variables had their coefficients shrunk to zero, suggesting that after regularization, they contributed little additional predictive value beyond the selected factors.

**Table 3:** LASSO variable selection.

Variable	Coeficient (β) at λ_1SE	Odds Ratio [exp(β)]	Selected (Yes/No)
Age over 35 years	0.15	1.16	Yes
BMI over 30 kg/m2	0.06	1.06	Yes
History of GDM	0.11	1.11	Yes
History of Hypertension	–	–	No
Family history of diabetes	–	–	No
History of abortion	–	–	No
History of dyslipidemia	–	–	No
History of PCOS	–	–	No
History of macrosomia	–	–	No
History of fetal anomalia	–	–	No
History of IUFD	–	–	No

GDM, Gestational Diabetes Mellitus; BMI, Body Mass Index; PCOS, Polycystic Ovary Syndrome; IUFD, Intrauterine Fetal Death .

**Figure 2:**
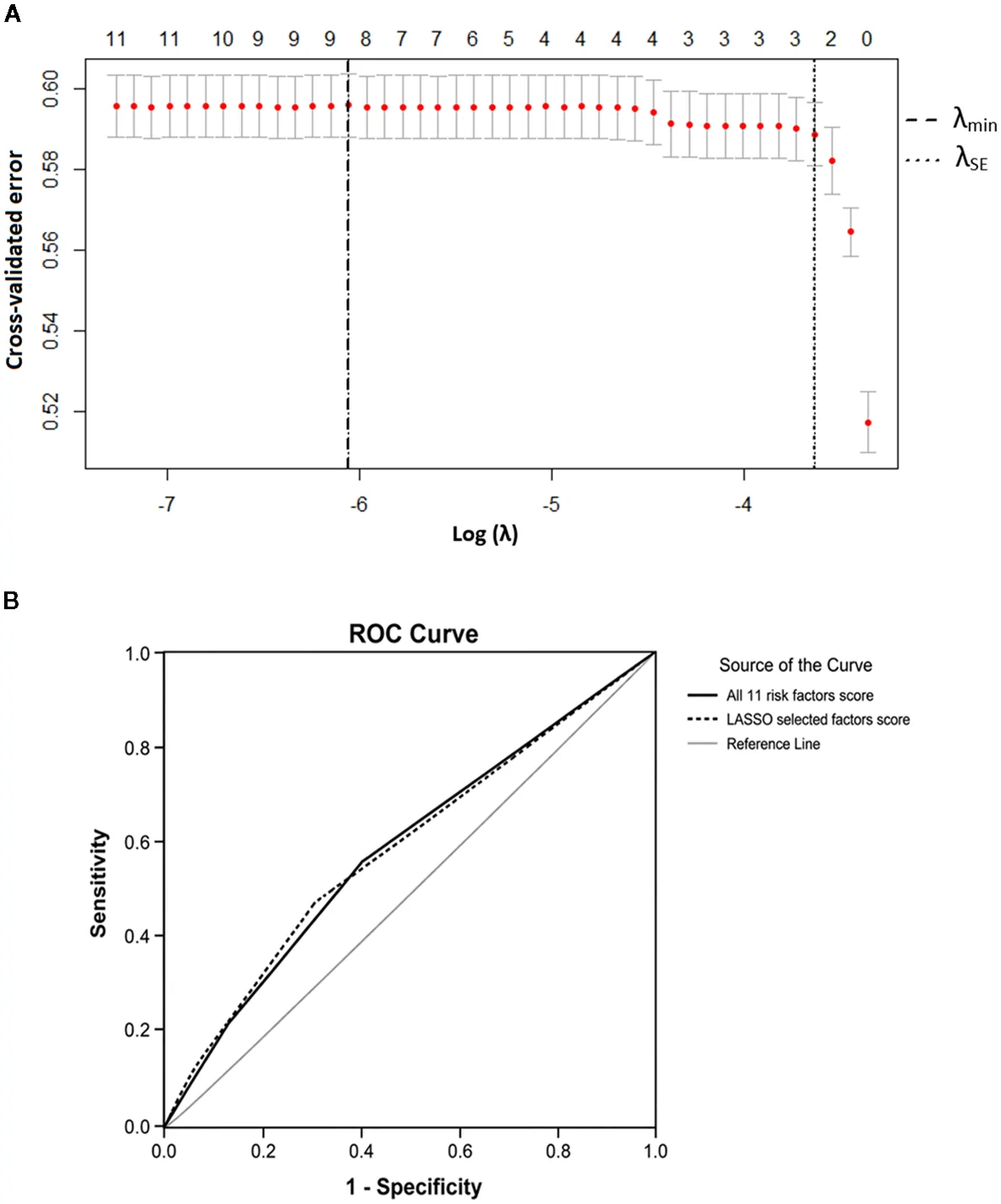
Variable selection of LASSO-based model and comparison with full 11-factor model for prediction of gestational diabetes mellitus (GDM). **(A)** Cross validation plot for LASSO regression model for potential maternal risk factors for GDM. **(B)** Receiver operating characteristic (ROC) curves comparing the predictive performance of the full model (including all maternal risk factors) and the parsimonious model derived using LASSO for the prediction of GDM.

The results of the LASSO regression with 10-fold cross-validation are presented in **Figure 2**.A. Using the more conservative λ_1_SE criterion, the final model retained three predictors: maternal age > 35 years, BMI ≥ 30 kg/m^2^, and a history of previous GDM. These variables demonstrated the most stable and robust contribution to GDM prediction after regularization. Although a larger number of maternal characteristics showed associations with GDM at lower penalty levels, only these three predictors remained consistently influential when model complexity was constrained to enhance generalizability. This finding suggests that advanced maternal age, obesity, and prior GDM history represent the core maternal risk profile for GDM in this population, as also reflected in the multivariable results presented in **Table 3**.

The ROC Curves Comparing Predictive Performance of Full vs. LASSO Parsimonious Models for GDM Prediction are presented in **Figure 2**. The results indicated that the simpler model incorporating only the three key predictors selected by LASSO (AUC≈0.59) performed nearly identically to the more complex model that included all eleven variables (AUC≈0.58). Consequently, the model was substantially simplified without any detectable loss of predictive performance.

### 3.4 Performance comparison of two screening models

The comparison of GDM detection rates between full 11-factor model and LASSO 3-factor model screening strategies is shown in **Table 4**. In the Full 11-Factor Model, 20,334 individuals were not eligible for GDM screening, of whom 2223 (10.9%) developed GDM during their pregnancy. Conversely, the LASSO 3-factor model classified 23,599 individuals as ineligible for GDM screening, among whom 11.2% developed GDM. Among the individuals that were eligible for screening, the Full 11-Factor Model demonstrated a GDM detection rate of 18.6%. The LASSO 3-Factor Model showed improved effectiveness, achieving a detection rate of 20.2% among eligible screened individuals. This suggests that the model based on fewer factors was better at identifying cases of GDM among those who were screened.

**Table 4:** Comparison of GDM Detection Rates Between Full 12-Factor Model and LASSO 3-Factor Model Screening Strategies.

Model	GDM Screening	Number of eligible women	Number of GDM Cases (N=5030)	Proportion of GDM among individuals for whom screening is not recommended according to their risk profile.	Proportion of GDM among individuals for whom screening is recommended according to their risk profile.
Full 11-Factor Model	Not Recommended	20,334	2,223	10.9%	N/A
	Recommended	15,096	2807	N/A	18.6%
LASSO 3-Factor Model	Not Recommended	23,599	2,638	11.2%	N/A
	Recommended	11,831	2,392	N/A	20.2%

### 3.5 Perinatal adverse outcomes stratified by GDM and risk status

The distribution of pregnancy outcomes among four groups categorized by GDM status and the presence of maternal risk factors is shown in **Supplementary Table 1**. Compared with the reference group (non-GDM without maternal risk factor), women in Group 2 (non-GDM with maternal risk factor) and Group 4 (GDM with maternal risk factor) showed a higher frequency of adverse outcomes including macrosomia, preterm birth, preeclampsia, hyperbilirubinemia, NICU admission, and IUFD (*P* < 0.001). In contrast, no significant differences were observed among the groups in terms of low birth weight. Although birth trauma remained relatively rare, its incidence was significantly higher in Group 4 compared with the reference group (*P* = 0.006).

The adjusted odds ratios (ORs) for adverse pregnancy outcomes across the study groups are presented in **Table 5**. Overall, both maternal risk factors and GDM were associated with increased odds of several complications, with the combined presence of GDM and maternal risk factors (Group 4) showing the strongest associations.

**Table 5:** Odds ratios (ORs) and 95% confidence intervals (CIs) for pregnancy outcomes according to GDM status and maternal risk factors.

Pregnancy outcome	Group levels (Ref: Group 1)	Model 1			Model 2		
		OR	95% CI	P-value	OR	95% CI	P-value
Macrosomia	Group 2	1.53	1.38-1.69	<0.001	1.04	0.89 – 1.20	0.638
	Group 3	1.13	0.92-1.68	0.231	1.04	0.80 – 1.34	0.741
	Group 4	2.56	2.22-2.94	<0.001	1.65	1.35 – 2.01	< 0.001
Preterm	Group 2	1.37	1.25 – 1.51	<0.001	1.38	1.20 – 1.59	< 0.001
	Group 3	1.15	0.95 – 1.38	0.152	1.06	0.82 – 1.34	0.647
	Group 4	1.64	1.41 – 1.90	<0.001	1.46	1.19 – 1.79	< 0.001
Preeclampsia	Group 2	1.43	1.32 – 1.54	<0.001	1.12	1.01 – 1.25	0.034
	Group 3	1.16	1.00 – 1.35	0.049	1.09	0.91 – 1.31	0.343
	Group 4	1.67	1.48 – 1.88	<0.001	1.23	1.05 – 1.44	0.010
Hypoglycemia	Group 2	1.12	0.62 – 1.98	0.709	0.87	0.43 – 1.72	0.683
	Group 3	15.43	9.73 – 25.02	<0.001	17.67	10.41 – 30.97	< 0.001
	Group 4	48.41	32.87 – 74.23	<0.001	41.91	25.22 – 72.68	< 0.001
Hypocalcemia	Group 2	1.11	0.62 – 1.94	0.728	0.79	0.40 – 1.53	0.495
	Group 3	10.93	6.70 – 18.04	<0.001	10.99	6.40 – 19.07	< 0.001
	Group 4	25.35	16.93 – 39.23	<0.001	18.48	10.85 – 32.24	< 0.001
Hyperbilirubin	Group 2	1.24	1.13 – 1.36	<0.001	1.42	1.25 – 1.60	< 0.001
	Group 3	1.00	0.83 – 1.20	0.966	0.98	0.78 – 1.22	0.870
	Group 4	1.48	1.28 – 1.71	<0.001	1.57	1.30 – 1.89	< 0.001
NICU	Group 2	1.39	1.25 – 1.55	<0.001	1.25	1.07 – 1.46	0.005
	Group 3	1.49	1.22 – 1.81	<0.001	1.56	1.23 – 1.97	< 0.001
	Group 4	2.41	2.07 – 2.80	<0.001	2.16	1.76 – 2.66	< 0.001
Birth trauma	Group 2	1.30	0.96 – 1.78	0.092	1.08	0.66 – 1.77	0.752
	Group 3	0.95	0.46 – 1.74	0.871	1.27	0.51 – 2.70	0.563
	Group 4	1.88	1.18 – 2.90	0.006	1.80	0.92 – 3.38	0.076
LBW	Group 2	1.08	1.00 – 1.17	0.061	1.17	1.03 – 1.32	0.018
	Group 3	0.91	0.77 – 1.07	0.266	1.04	0.84 – 1.28	0.711
	Group 4	1.01	0.87 – 1.16	0.929	0.98	0.80 – 1.20	0.858
IUFD	Group 2	1.58	1.20 – 2.09	0.001	1.73	1.10 – 2.70	0.016
	Group 3	0.91	0.46 – 1.63	0.778	1.56	0.70 – 3.12	0.240
	Group 4	1.85	1.19 – 2.78	0.004	2.39	1.27 – 4.36	0.005

Group 1, non-GDM without maternal risk factor; Group 2, non-GDM with maternal risk factor; Group 3, GDM without maternal risk factor; Group 4, GDM with maternal risk factor.

Model 1: Crude model, Model 2: Adjusted model for age, BMI, gestational age at enrollment, gestational age at delivery, GDM screening protocol and delivery type.

## 4 Discussion

In this large analysis of 35,430 pregnancies, approximately 11% of women who were not recommended for GDM screening would have had undiagnosed GDM under risk factor-based targeted screening strategies; however, the full 11-factor model and the simplified three-factor LASSO model demonstrated comparable performance. Notably, women diagnosed with GDM who also exhibited established maternal risk factors had a significantly greater likelihood of adverse pregnancy outcomes compared with women with GDM but without identifiable maternal risk factors. Furthermore, women with GDM in the absence of maternal risk factors demonstrated a lower overall rate of adverse outcomes than non-GDM women who possessed maternal risk factors, although their risk remained slightly higher than that observed among non-GDM women without maternal risk factors.

GDM is associated with significant maternal and neonatal morbidity (19). Evidence from large studies, including the Hyperglycemia and Adverse Pregnancy Outcome (HAPO) study, demonstrates that increasing maternal glycemia is linked to higher risks of preeclampsia, cesarean delivery, fetal overgrowth, and neonatal complications such as hypoglycemia and NICU admission (20). Women with GDM have a 1.5–3-fold increased risk of several adverse outcomes, underscoring the importance of effective screening and timely management; screening strategies that fail to catch many affected pregnancies may lead to significant consequences (21).

Worldwide, there is no agreement on the best way to screen GDM, and recommendations differ widely among professional organizations. The American Diabetes Association (ADA) (2) and the International Association of Diabetes and Pregnancy Study Groups (IADPSG) (22) advocate for universal screening with a one-step 75-g oral glucose tolerance test (OGTT), while the American College of Obstetricians and Gynecologists (ACOG) supports a two-step approach (first a 50-g glucose challenge test, then a 100-g OGTT) (6). Meanwhile, some European regions and less-resourced settings continue to favor selective screening based on maternal risk factors to lower costs and reduce laboratory burden (23). However, research indicates that risk factor-based screening may fail to detect a substantial proportion of GDM cases, 20% to 50% of GDM cases, depending on the criteria used and the population involved (8, 24). In addition, there is considerable variation across clinical guidelines in how GDM risk factors are defined, including differences in thresholds for maternal age, body mass index, height, and obstetric history (25–27). As a result, the proportion of pregnant individuals classified as high risk can range from about 40% to over 70%, which limits the discriminatory value and practicality of selective screening strategies (28).

Cost perspective, analyses comparing universal and risk-based screening have also produced inconsistent results, with studies differing on whether limiting testing reduces costs or whether universal screening is more cost-effective due to earlier detection and prevention of complications (10, 14, 29). Although selective screening may reduce initial testing expenditures, several economic and modeling studies suggest that universal screening may be more cost-effective when long-term maternal and neonatal outcomes are considered. Earlier detection and treatment of GDM through universal screening have been associated with reductions in adverse outcomes such as fetal macrosomia, cesarean delivery, neonatal intensive care unit admissions, and birth-related complications, as well as a lower future risk of type 2 diabetes and other metabolic disorders in affected mothers (30, 31).

In high-income settings, the cost of universal screening generally falls within accepted pay thresholds, particularly in populations where the prevalence of GDM exceeds approximately 5–7%. These findings suggest that the economic justification for selective screening may be weaker than previously assumed, especially in populations with moderate to high GDM prevalence where broader screening may yield greater clinical and economic benefits (32–34).

The application of LASSO regression provided important insights into the structure of GDM risk prediction. By penalizing weak or unstable coefficients, the analysis identified a parsimonious model consisting of three key predictors, advanced maternal age, elevated BMI, and a prior history of GDM, which captured nearly all of the predictive information contained within the broader clinical risk profile. Accordingly, the present study found that the simplified three-factor LASSO model demonstrated predictive performance comparable to that of the full 11-factor model. In contrast, several traditionally recognized secondary risk factors, including family history of diabetes and PCOS, were reduced to negligible contributions in the penalized model, suggesting substantial collinearity with maternal age and adiposity. However, the relatively modest AUC observed even in the optimized model highlights an important biological limitation of risk factor–based prediction. The pathophysiology of GDM is largely driven by complex placental hormonal and endocrine changes during pregnancy, which cannot be fully captured using pre-pregnancy maternal characteristics alone (35). In addition, the profound metabolic adaptations that occur throughout pregnancy may outweigh baseline risk profiles, resulting in a substantial proportion of GDM cases developing in women without traditional clinical risk factors (35–37).

Furthermore, the outcome analysis provides important context regarding the broader impact of underlying metabolic vulnerability. As expected, women with both GDM and baseline risk factors had the highest likelihood of adverse perinatal outcomes. However, women who possessed baseline risk factors but did not receive a GDM diagnosis also demonstrated significantly elevated risks compared with those without such factors. This finding suggests that the metabolic disturbances associated with advanced maternal age and obesity may independently contribute to adverse pregnancy outcomes, in addition to the transient hyperglycemia characteristic of GDM. While targeted screening strategies are designed to improve resource allocation, they may underestimate the compounded risk that arises when underlying metabolic vulnerability coexists with undetected GDM.

A key strength of this study was the use of LASSO regression, which allowed for the selection of relevant variables while preventing overfitting (38). By applying an L1 penalty, LASSO reduced weak or unstable coefficients towards zero and kept only the most informative predictors, addressing common issues found in traditional logistic regression. This method improved model stability and generalizability, identifying a clear set of relevant risk factors that can be implemented in real-world screening practices. Additionally, our study benefited from a large sample size, population-based design, and the use of routinely collected prenatal data, which strengthen the validity of our findings. By directly comparing risk-based screening strategies with observed GDM outcomes, rather than relying solely on theoretical models or simulated projections, our analysis provides practical insight into the real-world implications of selective screening in routine clinical practice.

However, several limitations should be considered when interpreting the findings of this study. First, as this investigation represents a secondary analysis of data derived from a previously conducted trial, the scope of the analysis was inherently constrained by the variables collected in the original study protocol. Consequently, potentially relevant clinical, biochemical, and socioeconomic factors that may influence the risk and diagnosis of GDM, such as detailed dietary habits, physical activity, and specific markers of insulin resistance, were unavailable for evaluation. In addition, the retrospective nature of secondary analyses limits the ability to establish causal relationships and may introduce residual confounding despite statistical adjustment. Moreover, the risk factor-based screening strategies evaluated here were simulated using baseline characteristics, rather than being executed in routine clinical practice. Although this allowed for a direct comparison with universal screening, real-world implementation may involve clinical judgment and contextual factors that could affect screening accuracy. Furthermore, the cohort consisted of women from various centers in Iran and was relatively uniform regarding healthcare organization and risk profiles. This limits the generalizability of our findings to populations with different ethnic backgrounds, obesity rates, diabetes risk, or healthcare systems. In addition, this study did not evaluate the cost-effectiveness of different screening strategies or examine long-term maternal and offspring outcomes, including postpartum diabetes, future cardiovascular risk, and childhood metabolic health. Consequently, the broader clinical and economic implications of selective versus universal screening could not be fully assessed. Future research should therefore investigate the impact of different screening approaches on both short- and long-term maternal and neonatal outcomes, while also evaluating healthcare resource utilization and cost-effectiveness across diverse populations and healthcare settings.

## 5 Conclusion

In this large population-based cohort, we found that screening strategies based solely on predefined maternal risk factors would fail to identify a proportion of women with GDM. Although the simplified three-factor LASSO model demonstrated predictive performance comparable to that of the full 11-factor model, reliance on risk factor–based screening alone remained insufficient for identifying all affected pregnancies. Importantly, maternal risk factors were strongly associated with adverse pregnancy outcomes independent of GDM diagnosis. Women with both GDM and established maternal risk factors had the highest likelihood of adverse outcomes. In contrast, women with GDM but without maternal risk factors experienced lower rates of complications than non-GDM women who possessed maternal risk factors, although their risk remained slightly higher than that of women without either condition. These findings suggest that the coexistence of GDM and underlying maternal risk factors confers an additional level of clinical vulnerability and warrants closer monitoring.

Overall, our results indicate that reliance on selective, risk factor–based screening alone is unlikely to effectively identify all pregnancies at risk for GDM or its related complications. Universal screening therefore remains the most reliable approach for timely detection and management in populations with similar risk profiles. Nevertheless, the simplified three-factor model may offer a practical alternative for initial risk assessment in resource-limited settings, where comprehensive risk evaluation or universal testing may be less feasible, as it reduces the number of variables required and facilitates rapid clinical assessment. Maternal risk factors should also be recognized as important indicators of pregnancy risk, even in the absence of a formal GDM diagnosis.

## Data Availability

The raw data supporting the conclusions of this article will be made available by the authors, without undue reservation.
