# Supplementary material for: Universal versus risk– factor–based screening for gestational diabetes mellitus: findings from a large–scale cohort study
Source: Front Endocrinol (Lausanne). 2026 Aug 13;17:1918348. doi: 10.3389/fendo.2026.1918348 (PMC13518169; doi:10.3389/fendo.2026.1918348)
Supplement: Supplementary file 1 [file Table1.docx]

| Supplementary Table 1. Distribution and comparison of pregnancy outcomes among four groups defined by GDM status and presence of maternal risk factors | | | | | | | | |
| --- | --- | --- | --- | --- | --- | --- | --- | --- |
| Pregnancy outcomes | | Groups N (%) | | | | Tests | | |
|  |  | Group 1:  n=18111 | Group 2:  n=12289 | Group 3:  n=2223 | Group 4:  n=2807 | *P*-value ^*^ | *P*-value ** | *P*-value *** |
| Macrosomia | No | 16564 (95.4) | 10732 (93.2) | 2020 (94.8) | 2375 (89.1) | <0.001 | 0.252 | <0.001 |
|  | Yes | 796 (4.6) | 789 (6.8) | 110 (5.2) | 292 (10.9) |  |  |  |
| Preterm | No | 16340 (94.6) | 10683 (92.7) | 1999 (93.8) | 2439 (91.5) | <0.001 | 0.158 | <0.001 |
|  | Yes | 933 (5.4) | 837 (7.3) | 131 (6.2) | 228 (8.5) |  |  |  |
| Preeclampsia | No | 16481 (91.4) | 10841 (88.2) | 2005 (90.2) | 2427 (86.5) | <0.001 | 0.051 | <0.001 |
|  | Yes | 1543 (8.6) | 1448 (11.8) | 218 (9.8) | 380 (13.5) |  |  |  |
| Hypoglycemia | No | 17333 (99.8) | 11500 (99.8) | 2080 (97.7) | 2480 (93) | 0.766 | <0.001 | <0.001 |
|  | Yes | 27 (0.2) | 20 (0.2) | 50 (2.3) | 187 (7) |  |  |  |
| Hypocalcemia | No | 18083 (99.8) | 12268 (99.8) | 2186 (98.3) | 2701 (96.2) | 0.772 | <0.001 | <0.001 |
|  | Yes | 28 (0.2) | 21 (0.2) | 37 (1.7) | 106 (3.8) |  |  |  |
| Hyper bilirubin | No | 15852 (93.5) | 10476 (92) | 1954 (93.4) | 2386 (90.6) | <0.001 | 0.956 | <0.001 |
|  | Yes | 1107 (6.5) | 908 (8) | 137 (6.6) | 247 (9.4) |  |  |  |
| NICU | No | 17415 (96.2) | 11642 (94.7) | 2098 (94.4) | 2560 (91.2) | <0.001 | <0.001 | <0.001 |
|  | Yes | 696 (3.8) | 647 (5.3) | 125 (5.6) | 247 (8.8) |  |  |  |
| Birth trauma | No | 18025 (99.5) | 12213 (99.4) | 2213 (99.6) | 2782 (99.1) | 0.093 | 0.518 | 0.006 |
|  | Yes | 86 (0.5) | 76 (0.6) | 10 (0.4) | 25 (0.9) |  |  |  |
| LBW | No | 15448 (91.1) | 10295 (904) | 1920 (91.8) | 2397 (91) | 0.062 | 0.270 | 0.941 |
|  | Yes | 1511 (8.9) | 1089 (9.6) | 171 (8.2) | 236 (9) |  |  |  |
| IUFD | No | 18013 (99.5) | 12184 (99.1) | 2212 (99.5) | 2779 (99) | 0.001 | 0.878 | 0.005 |
|  | Yes | 98 (0.5) | 105 (0.9) | 11 (0.5) | 28 (1) |  |  |  |
| Group 1: non-GDM without maternal risk factor; Group 2: non-GDM with maternal risk factor; Group 3: GDM without maternal risk factor; Group 4: GDM with maternal risk factor  *: Group 2 versus 1, **: Group 3 versus 1, ***: Group 4 versus 1 | | | | | | | | |
|  | | | | | | | | |
